# Disruption of the Suprachiasmatic Nucleus in Fibroblast Growth Factor Signaling-Deficient Mice

**DOI:** 10.3389/fendo.2016.00011

**Published:** 2016-02-08

**Authors:** Ann V. Miller, Scott I. Kavanaugh, Pei-San Tsai

**Affiliations:** ^1^Department of Integrative Physiology, University of Colorado Boulder, Boulder, CO, USA

**Keywords:** SCN, *Fgf8*, *Fgfr1*, development, cFos, VIP

## Abstract

Fibroblast growth factor (Fgf) 8 is essential for the development of multiple brain regions. Previous studies from our laboratory showed that reduced Fgf8 signaling led to the developmental alterations of neuroendocrine nuclei that originated within the diencephalon, including the paraventricular (PVN) and supraoptic (SON) nuclei. To further understand the role of Fgf8 in the development of other hypothalamic nuclei, we examined if Fgf8 and its cognate receptor, Fgfr1, also impact the integrity of the suprachiasmatic nuclei (SCN). The SCN control an organism’s circadian rhythm and contain vasoactive intestinal peptide (VIP)-producing neurons as the main input neurons. Mice hypomorphic for *Fgf8*, *Fgfr1*, or both were examined for their SCN volume and the number of VIP neurons on postnatal day (PN) 0; adult hypomorphic mice were further examined for SCN function by quantifying SCN neuronal activation using cFos as a marker. On PN0, mice homozygous for *Fgf8* hypomorphy displayed the most severe reduction of the SCN volume and VIP neurons. Those heterozygous for *Fgf8* hypomorphy alone or *Fgf8* combined with *Fgfr1* hypomorphy, called double heterozygotes (DH), showed normal SCN volume but significantly reduced VIP neurons, albeit less severely than the homozygotes. Adult wild type, heterozygous *Fgf8* hypomorphs (F8 Het), and DH mice were also examined for SCN cFos activation at three time points: 1 h (morning), 6 h (afternoon), and 11 h (evening) after light onset. In F8 Het mice, a significant change in the pattern of cFos immunostaining that may reflect delayed morning SCN activation was observed. Overall, our studies provide evidence supporting that deficiencies in *Fgf8* not only impact the structural integrity of the SCN but also the pattern of SCN activation in response to light.

## Introduction

In early development, the ventral diencephalon undergoes significant differentiation to form the neuroendocrine hypothalamus. The hypothalamus controls crucial biological functions such as metabolism, growth, stress, reproduction, and many more. The fibroblast growth factor (Fgf) signaling family consists of 22 ligands and 4 tyrosine kinase receptors and is known to control the development of the brain (1), and importantly, the hypothalamus itself (2). *Fgf8*, one of the Fgf ligands, is expressed in the anlagen of the hypothalamus by embryonic day (E) 9.5 in the developing mouse embryos (3), with particularly robust expression in regions surrounding the optic chiasm (4). *Fgfr1*, one of the cognate receptors of Fgf8, is also expressed in the developing mouse embryo along the linings of the third ventricle where multiple hypothalamic neurons emerge (5). The sites of *Fgf8* and *Fgfr1* expression suggest an important role of Fgf8 signaling through Fgfr1 in the birth and survival of multiple hypothalamic neuronal populations. Consistent with these observations, we previously demonstrated that disrupted Fgf8 signaling reduced hypothalamic oxytocin-producing neurons in the paraventricular (PVN) and supraoptic (SON) nuclei (6).

The suprachiasmatic nuclei (SCN) of the hypothalamus consist of thousands of heterogeneous neurons that regulate endogenous circadian rhythms and are considered the master clock in mammals (7, 8). The paired nuclei are subdivided into two major regions: the ventrolateral core and the dorsomedial shell. The SCN receive photic stimuli from the retina via the retinohypothalamic tract (RHT) (9, 10). Direct innervation from specialized melanopsin-containing retinal ganglion cells also modulates the light entrainment of the SCN (11). Although the SCN generate endogenous circadian rhythms in the absence of external cues, they also respond to and entrain to exogenous cues such as the light/dark cycle. The SCN are resistant to resetting from non-photic cues that typically cause phase-shifts in peripheral clocks (12–14); thus, light is the main stimulus for the SCN. Once the light signal reaches the SCN, it will first activate the main input neurons producing vasoactive intestinal peptide (VIP) within the SCN core (15). Consequently, VIP, a 28-amino acid peptide, is released to control and maintain communication among other regions of the SCN and to generate continual SCN rhythmicity (16, 17). The expression of cFos, an immediate early gene, in the SCN neurons has been successfully used as a cellular marker for the photic activation of the SCN (18).

Because disrupted Fgf8 signaling reduces oxytocin-producing neurons in the PVN and SON (6), we hypothesize that other hypothalamic neuronal populations may also be markedly affected when Fgf signaling is impaired. We further hypothesized that deficiencies specifically in Fgf8 and one of its cognate receptors, Fgfr1, impact the development and the integrity of the SCN. Using newborn and adult transgenic mice hypomorphic for *Fgf8*, *Fgfr1*, or both, we examine if Fgf signaling deficiencies disrupt the SCN development and alter the pattern of SCN activation later in life. Our results reveal severe developmental disruptions of the SCN in newborn *Fgf8-* and *Fgfr1-*deficient pups. Further, gauged by cFos immunostaining, *Fgf8* deficiency significantly alters the pattern of SCN neuronal activation in adults during the light phase, supporting a critical role of Fgf8 in the organization of the SCN neurocircuit.

## Materials and Methods

### Animals

Mice hypomorphic for *Fgf8* [(19), 129P2/OlaHsd* CD-1, obtained from Mouse Regional Resource Centers, Davis, CA, USA] or hypomorphic for *Fgfr1* [(20), 129sv/CD-1, obtained from Canadian Mutant Mouse Repository] were kept on a 12L:12D photoperiod and fed water and rodent chow *ad libitum*. *Fgf8* and *Fgfr1* hypomorphs have global reductions in functional transcript levels of 54 and 66–80%, respectively, due to a neo-cassette insertion that creates false splice sites. The mice used in these studies were bred at the University of Colorado at Boulder by crossing male and female mice each hypomorphic for both *Fgf8* and *Fgfr1* alleles (called double heterozygous or DH mice). This cross produced offspring of seven genotypes: wild type (WT), *Fgf8* heterozygous hypomorph (F8 Het), *Fgf8* homozygous hypomorph (F8 Hom), *Fgfr1* heterozygous hypomorph (R1 Het), *Fgfr1* homozygous hypomorph (R1 Hom), DH, and double homozygous hypomorph (DHom). All homozygous animals, including F8 Hom, R1 Hom, and DHom, die within 24 h of birth and were used only for the study of postnatal day (PN) 0 animals. Adult WT, F8 Het, and DH mice were used to examine the neuronal activation within the SCN at different time points during the light phase. Genotypes of the animals were determined by polymerase chain reaction (PCR) of genomic DNA obtained from tail biopsies. All animal procedures complied with protocols approved by the Institutional Animal Care and Use Committee at the University of Colorado Boulder.

### Activation of cFos in Adult SCN

Mixed-sex adult mice between PN50 and PN60 were used to examine the activation of cFos in the SCN. WT, F8 Het, and DH mice were collected at three different time points throughout the day: 1 h (morning), 6 h (afternoon), and 11 h (evening) after the onset of light. After isoflurane anesthesia, mouse brains were harvested and blocked at 0.50 and −2.06 mm anterior and posterior of bregma, respectively, immersion-fixed in 4% paraformaldehyde at 4°C for 24 h, and cryoprotected in 30% sucrose until cFos immunohistochemistry (IHC).

### VIP and cFos Immunohistochemistry

For VIP immunostaining, brains of mixed-sex PN0 pups were harvested at 2 h after light onset, immersion-fixed in 4% paraformaldehyde at 4°C for 6 h, cryoprotected in 30% sucrose, coronally sectioned at 20-μm thickness using a cryostat, and thaw-mounted onto gelatin-coated slides. For cFos immunostaining, adult brains described above were cryosectioned at 50-μm thickness, and floating sections were collected into phosphate-buffered saline (PBS). IHC for VIP was performed using an anti-VIP antibody [1:1000; a gift from Dr. Dick Swaab (21–23)], and IHC for cFos was performed using an anti-cFos antibody [1:400; Santa Cruz Biotechnology, Dallas, TX, USA (24–26)]. Sections were incubated in the primary antibody for 1 week (for VIP IHC) or 48 h (for cFos IHC). The sections were then incubated sequentially with a secondary biotinylated donkey anti-rabbit antibody, avidin–biotin complex (ABC; Vector Labs, Burlingame, CA, USA), and reacted with diaminobenzidine with (cFos IHC) or without (VIP IHC) 0.05% nickel enhancement for color detection. After the color reaction, floating sections were washed and mounted onto gelatin-coated slides. All slides were counterstained with 1% methyl green, a nuclear stain, to visualize the perimeters of the SCN before dehydration and coverslipping.

### Quantification of SCN Neurons and Volume

The numbers of VIP- and cFos-immunoreactive (ir) neurons were counted by an investigator blind to the genotype of the animal. For VIP neuronal quantification, only SCN cells with clearly defined morphology and cytosolic VIP immunostaining were scored. For cFos quantification, an ocular grid was used to assist with the scoring. Only SCN cells with clear nuclear cFos immunostaining were scored. Because cFos staining intensity within the SCN was clearly bimodal, we also classified cFos-ir neurons subjectively into darkly and lightly stained neurons. We validated this classification by analyzing the optical density of 40 randomly selected c-Fos-ir neurons and found that the density of lightly stained neurons was 58.8 ± 1% of darkly stained neurons. For most analyses, all sections of the SCN were scored and summed to obtain the total neuron numbers positive for VIP or cFos. For the comparison of cFos staining between the SCN core and shell, only the six most medial SCN sections with a clear delineation of the core and shell were quantified. The SCN surface area was measured by freehand tracing of the SCN perimeter using NIH ImageJ. The SCN area in each section was calibrated and multiplied by the section thickness to obtain the volume. SCN volumes in all sections were summed to obtain the total volume of the SCN for each animal.

### Statistical Analysis

All statistical analyses were performed using Prism (GraphPad, La Jolla, CA, USA). PN0 data were analyzed using one-way ANOVA, and data from adults were analyzed by two-way ANOVA, both followed by Tukey’s multiple comparisons test. All data have been tested for normality using Bartlett’s test or Brown–Forsythe’s test. Differences were considered significant when *p* < 0.05.

## Results

### VIP Neurons and SCN Volume in PN0 Transgenic Mice Deficient in Fgf Signaling

**Figure 1:**
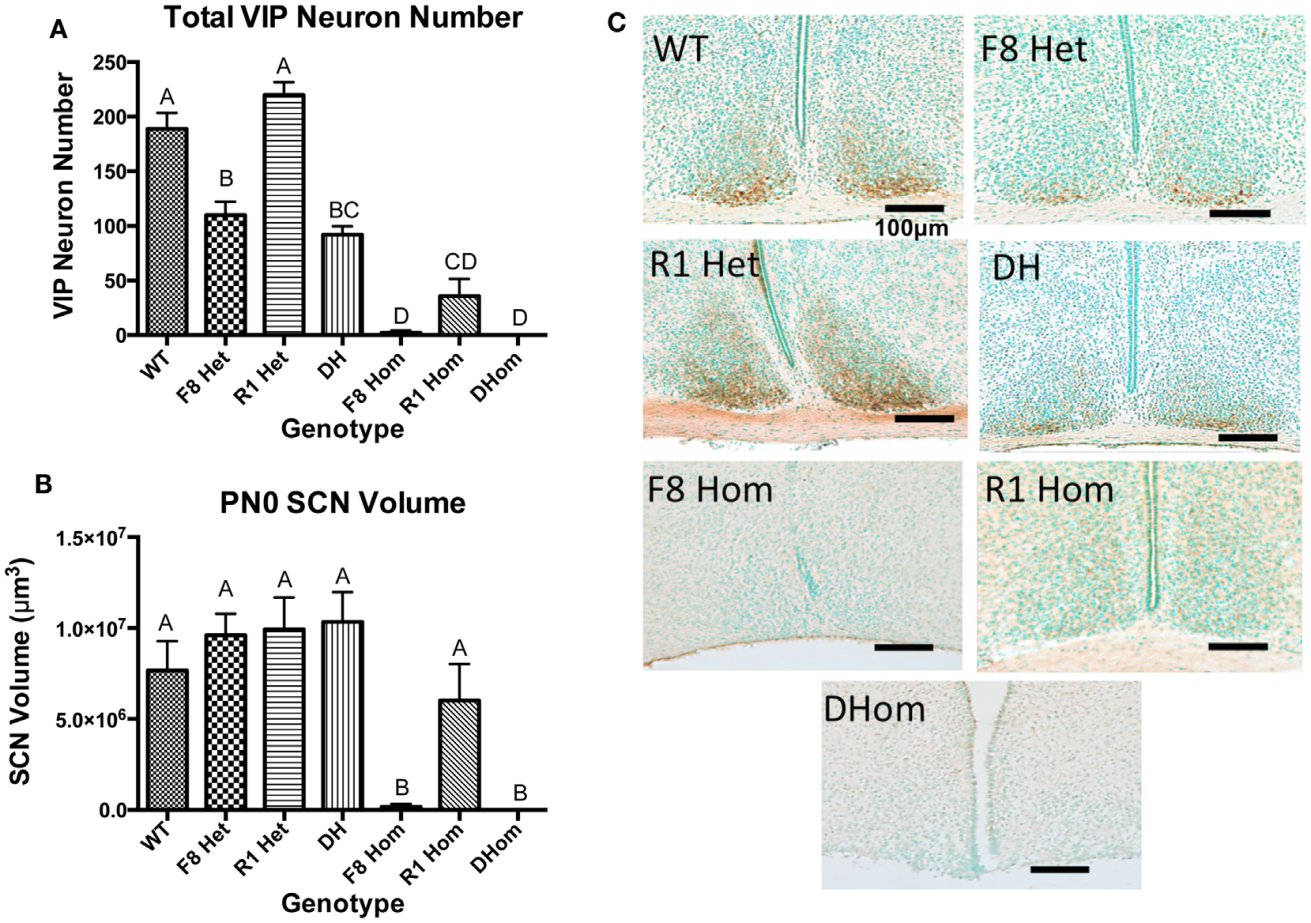
**(A)** VIP-ir neuron numbers in the SCN core of PN0 mice (WT, *n* = 6; F8 Het, *n* = 5; R1 Het, *n* = 5; DH, *n* = 5; F8 Hom, *n* = 4; R1 Hom, *n* = 3; Dhom, *n* = 3). Different letters indicate significant differences (*p* < 0.0001). **(B)** Total SCN volume at PN0. Different letters indicate significant differences (*p* < 0.0001). **(C)** Representative photomicrographs of VIP-ir neurons in the SCN at PN0. Scale bar = 100 μm. The brown staining indicates VIP-ir neurons, and green staining is the methyl green nuclear counterstain.

VIP neurons within the SCN core were counted in newborn pups across all seven genotypes. One-way ANOVA revealed a significant effect of genotype on VIP neurons [*F*(6,24) = 52.9, *p* < 0.001]; Figure 1A. Tukey’s multiple comparisons *post hoc* test revealed that except for R1 Het, all transgenic genotypes exhibited significantly reduced VIP neurons compared to WT animals (Figure 1A). All three homozygous mice (F8 Hom, R1 Hom, Dhom) had very few VIP neurons within the SCN core, but the two transgenic mice harboring homozygous *Fgf8* hypomorphy (F8 Hom and DHom) had the greatest deleterious effect. We further examined if the volume of the SCN in PN0 pups was altered by Fgf signaling deficiency (Figure 1B). One-way ANOVA revealed a significant effect of genotype on SCN volume [*F*(6,24) = 7.662, *p* = 0.0001]. Tukey’s multiple comparisons *post hoc* test revealed that F8Hom and DHom pups exhibited severely diminished SCN volume; however, other transgenic genotypes were not different from WT (Figure 1B). Representative photomicrographs of SCN VIP immunostaining in PN0 mice were shown in Figure 1C.

### SCN Volume in Adult Mice Deficient in Fgf Signaling

Because mice harboring homozygous hypomorphy in *Fgf8* and/or *Fgfr1* die within 24 h of birth and R1 Het mice did not exhibit any SCN-related phenotype on PN0, only WT, F8 Het, and DH mice were used for further adult studies. First, we analyzed the volume of adult SCN to examine if any age-dependent deterioration occurred in transgenic mice. The measurements of the SCN volume were 3.59 × 10^7^ ± 2.17 × 10^6^ μm^3^ for WT, 3.33 × 10^7^ ± 2.34 × 10^6^ μm^3^ for F8 Het, and 3.44 × 10^7^ ± 1.72 × 10^6^ μm^3^ for DH. Similar to PN0 data (Figure 1B), one-way ANOVA revealed no significant difference in the SCN volume among adult WT, F8 Het, and DH mice [*F*(11,60) = 0.8863, *p* = 0.5569].

### Total cFos Activation in SCN of Adult Mice Deficient in Fgf Signaling

**Figure 2:**
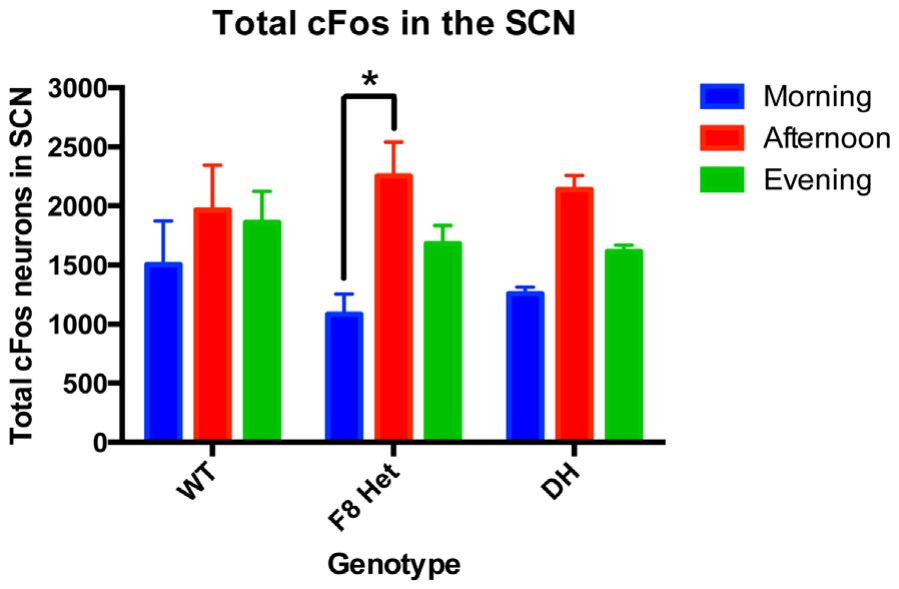
cFos-ir neurons in the entire SCN of PN 50–60 mice at three different time points: morning (1 h after the onset of light), afternoon (6 h after the onset of light), and evening (11 h after the onset of light). *N* = 4 for each genotype and time point. *p < 0.05.

The significant reductions of VIP neurons in PN0 F8 Het and DH mice (Figure 1A) suggest developmental abnormalities and possibly functional defects later in life. To address the latter, cFos IHC was performed on adult WT, F8 Het, and DH at 1, 6, and 11 h after light onset to gauge cFos activation in the presence of light (Figure 2). Two-way ANOVA revealed a significant effect of time of day [*F*(2, 27) = 9.445, *p* = 0.0008] but not genotype [*F*(2, 27) = 0.1995, *p* = 0.8204] on the number of SCN cFos-ir neurons. There was no significant genotype × time of day interaction [*F*(4, 27) = 0.6366, *p* = 0.6408]. Interestingly, F8 Het mice exhibited significantly elevated afternoon activation of cFos compared to the morning (*p* < 0.05), a feature lacking in both WT and DH mice, suggesting altered SCN activation in these mice.

**Figure 3:**
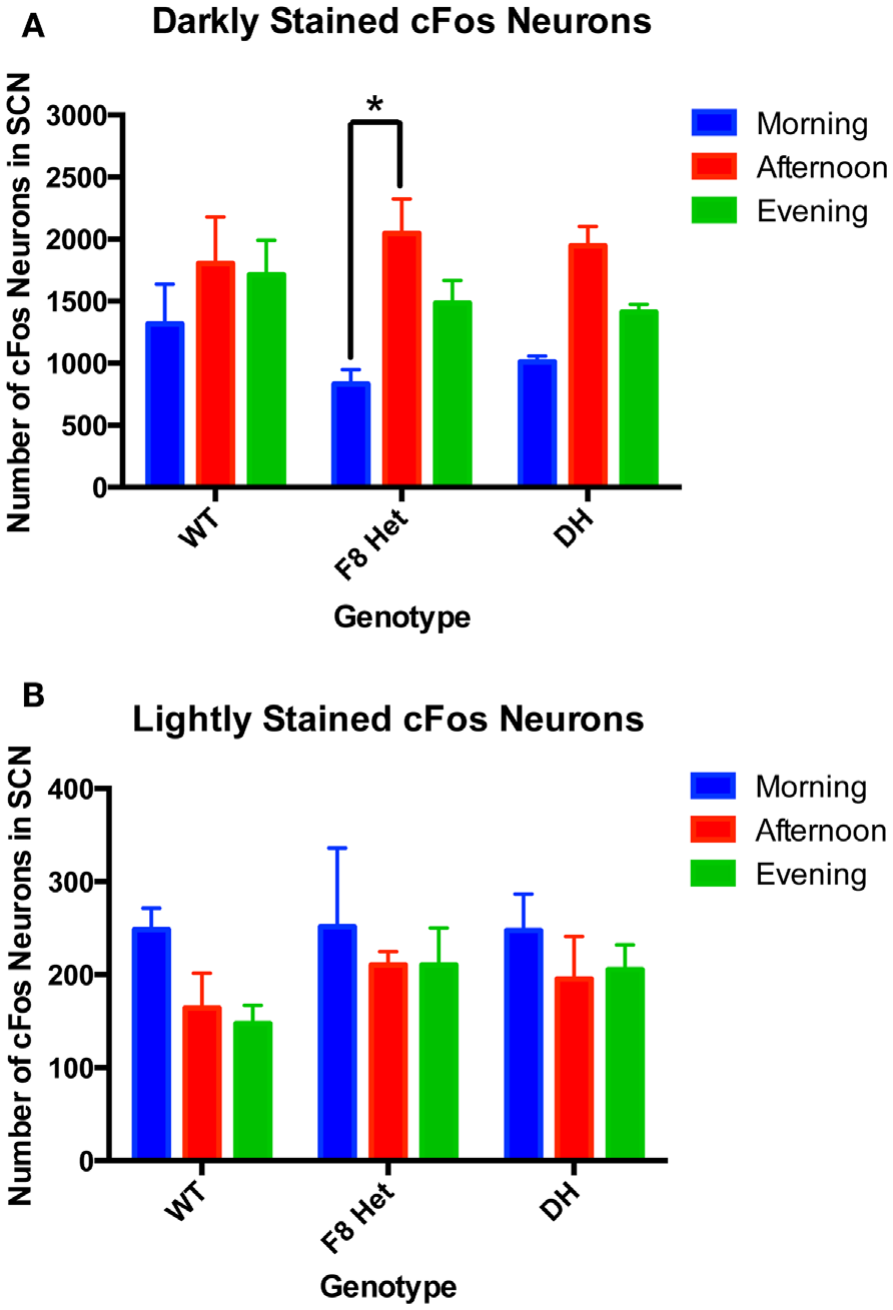
**(A)** Darkly stained and **(B)** lightly stained cFos-ir neuron numbers in the SCN of PN50-60 WT, F8 Het, and DH at three different time points: morning (1 h after the onset of light), afternoon (6 h after the onset of light), and evening (11 h after the onset of light). *N* = 4 for each genotype and time point. *p < 0.05.

Since the intensity of cFos staining may reflect the timing and degree of neuronal activation, we examined both darkly and lightly cFos-stained neurons among genotypes and at different time of day. Two-way ANOVA of neurons darkly stained for cFos (Figure 3A) showed a significant effect of time of day [*F*(2, 27) = 11.01, *p* = 0.0003] but no effect of genotype [*F*(2, 27) = 0.4640, *p* = 0.6337] or genotype × time of day interaction [*F*(4, 27) = 0.7137, *p* = 0.5898]. Specifically, the F8 Het animals displayed a significantly higher level of neuronal activation in the afternoon than morning (Figure 3A). Two-way ANOVA for lightly stained neurons (Figure 3B) showed no significant effect of genotype [*F*(2, 27) = 0.6740, *p* = 0.5181], time of day [*F*(4, 27) = 0.1796, *p* = 0.9470], or genotype × time of day interaction [*F*(4, 27) = 0.1796, *p* = 0.9470].

### Regional cFos Activation in SCN of Adult Mice Deficient in Fgf Signaling

**Figure 4:**
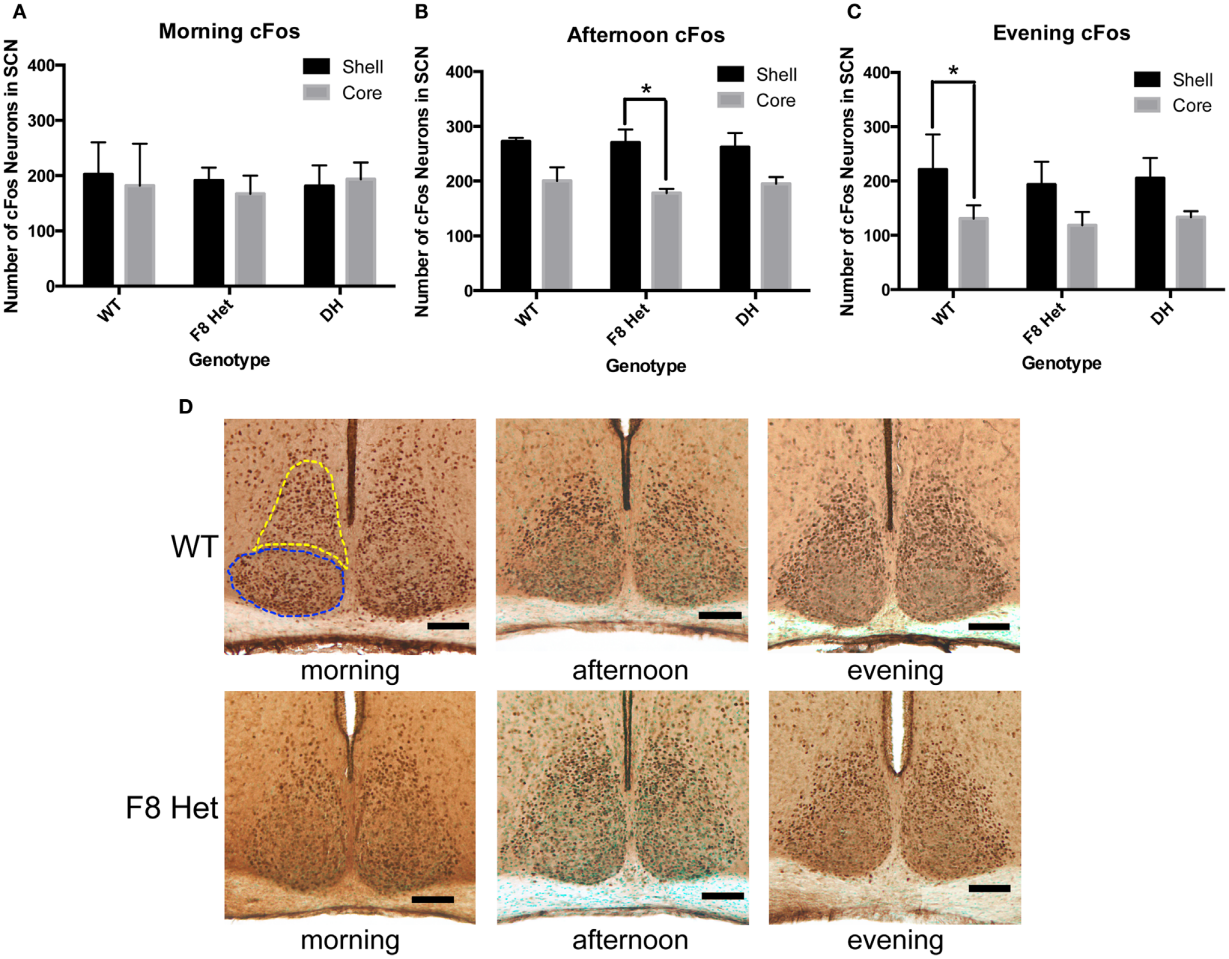
cFos-ir neurons within the SCN shell and the core in the **(A)** morning (1 h after the onset of light), **(B)** afternoon (6 h after the onset of light), or **(C)** evening (11 h after the onset of light). *N* = 4 for each genotype and time point. *p < 0.05. **(D)** Representative photomicrographs at each of the three time points for WT and F8 Het only. Blue dotted line outlines the core, and yellow dotted line outlines the shell.

We further investigated if cFos in the shell and the core of the SCN may be differentially activated among genotypes at different times of the day. In the morning, there was no effect of genotype or SCN subregion on cFos activation [*F*(2,18) = 0.09268; *p* = 0.9119, Figure 4A]. In the afternoon, although there was no genotype effect [*F*(2, 18) = 0.2176, *p* = 0.8065, Figure 4B], a significant effect of SCN subregion was observed [*F*(1, 18) = 25.68, *p* < 0.0001], with the *post hoc* test revealing a significant difference between the core and shell activation in only F8 Het (*p* < 0.05). There was no significant genotype × SCN subregion interaction in the afternoon [*F*(2, 18) = 0.2534, *p* = 0.7789, Figure 4B]. In the evening, there was no significant genotype effect [*F*(2, 18) = 0.5814, *p* = 0.5693, Figure 4C], but again, a significant effect of subregion was observed [*F*(1, 18) = 26.35, *p* < 0.0001], with the *post hoc* test revealing a significant difference between the core and shell activation in only WT (*p* < 0.05). There was no significant genotype × subregion interaction in the evening [*F*(2, 18) = 0.1320, *p* = 0.8772]. Figure 4D shows representative photomicrographs of SCN cFos immunostaining in WT and F8 Het mice.

## Discussion

In this study, we investigated if deficiencies in several Fgf signaling components impacted the structural development and adult function of the SCN. We demonstrated that at birth, mice with heterozygous deficiency in *Fgf8* (alone or combined with *Fgfr1*) had fewer VIP neurons compared to WT mice. Interestingly, the SCN were largely absent in mice with homozygous deficiency in *Fgf8* but not *Fgfr1*. Lastly, our data suggested Fgf8 deficiency altered the temporal pattern of SCN activation in adults. Overall, we have shown, for the first time, a significant role of Fgf signaling, in particular Fgf8, in establishing the structure and certain functional aspects of the SCN neurocircuit.

Several lines of evidence from the current study suggest *Fgf8* plays a more critical role than *Fgfr1* in the SCN development. First, in heterozygous transgenic pups, only those with *Fgf8* hypomorphy showed disrupted VIP neurons (Figure 1A). Heterozygous *Fgfr1* hypomorphy had no effect. Second, in homozygous transgenic pups, only those with *Fgf8* hypomorphy (F8 Hom and DHom) showed severely diminished or absent SCN. Homozygous *Fgfr1* hypomorphy had no effect on SCN volume (Figure 1B). The greater dependence of the SCN development on Fgf8 could be explained by Fgf signaling redundancy and expression patterns. In rodents, hypothalamic nuclei are formed by the clustering of neurons born within and migrate from the proliferative ventricular zone of the diencephalon (2, 27). *Fgfr1*, *2*, and *3* are all expressed along this proliferative zone (28), but *Fgf8* is the only ligand expressed robustly in the ventral diencephalon near the presumptive SCN (3, 5, 29). Fgfr1, 2, and 3 may work collaboratively to support the genesis of multiple hypothalamic neuronal populations along the ventricular zone; thus, deficiency in only Fgfr1 may not severely impact neurogenesis. Once hypothalamic neurons are fate-specified and reach the presumptive SCN, their maturation and survival in the SCN may depend solely on Fgf8 since other compensatory factors may not be available. This could also explain why heterozygous defects in *Fgf8* and *Fgfr1* were not additive in their developmental impact; the consequence of Fgf8 deficiency would be more deleterious and likely to mask that of Fgfr1 deficiency. Regardless, our results speak to the exquisite sensitivity of the developing SCN to Fgf8.

Although significant reductions in the SCN VIP neurons were observed in F8 Het and DH mice, their SCN volumes were not altered (Figure 1B). One interpretation was that VIP neurons may still be present in the SCN but were unable to produce their hallmark neuropeptide due to disrupted transcription, translation, or post-translational processing of the VIP prohormone. In support of this, we previously showed that Fgf8 deficiency delayed the processing of the oxyphysin prohormone in the PVN, leading to reduced oxytocin-ir neurons in newborn mice (6). Alternatively, VIP neurons may have died developmentally, but the loss of a few hundred neurons may not have a discernible impact on the total SCN volume.

We used cFo*s* as a marker of neuronal activation (18, 30) to gauge the pattern of SCN neuronal activation immediately after light onset (morning) and then throughout the remaining light phase (Figure 2). Although WT mice showed consistent levels of cFos activation throughout the day, F8 Het exhibited a significant increase in cFos activation in the afternoon compared to morning (Figure 2). The discrepant activation pattern could be due to a delayed morning cFos activation in F8 Het, leading to a greater difference between morning and afternoon. DH mice also exhibited a pattern similar to F8 Het, but a significant time difference was not observed.

The cFos activation in the SCN of our WT mice was consistent throughout different times of the day (Figure 2). Although similar results have been reported (31), there was considerable variability in the literature on the diurnal pattern of SCN cFos activation in rodents (32, 33). We surmise that differences in the time course examined and light regimen employed between the previous and current studies may underlie these variabilities. Regardless, the significant alteration in the pattern of cFos activation in F8 Het mice suggested *Fgf8* played a role in not only the development of the SCN, but also its temporal pattern of activation during the light phase.

Our total cFos neuronal counting (Figure 2) included lightly stained neurons that could confound our data interpretation because they may represent cells weakly activated or past the stage of optimal activation. As such, we discriminated between neurons of two staining intensities: lightly stained and darkly stained (Figure 3). The lightly stained neurons were present at a much lower level than the darkly stained neurons, suggesting most neurons were optimally activated at the time points sampled. As expected, lightly stained neurons exhibited no discernible pattern of activation throughout the day or differences among genotypes (Figure 3B). Darkly stained neurons (Figure 3A), however, recapitulated the pattern of total neurons stained with cFos (Figure 2). Overall, these results confirmed that our early data on total cFos-positive neuronal count correctly captured the temporal pattern of SCN activation.

We next interrogated if the cFos activation pattern differed between the SCN subregions among the genotypes. The core neurons receive a photic signal from the RHT and relay the signal to the remaining SCN; thus, one would expect the shell to become activated later than the core (34). Previous studies have also shown that *Per* gene expression, which regulates circadian oscillations within the SCN, is delayed in response to a light pulse in the shell compared to the core (35–37). Indeed, our data showed that as the day progressed, the shell became generally more activated than the core, but the difference between the core and shell occurred earlier in F8 Het (Figure 4B), suggesting several possibilities. First, the activation of the SCN core may have decreased prematurely in F8 Het, causing an earlier subregional difference. Second, WT mice may have relayed the signal more robustly to the shell, thus maintaining a higher level of activation later in the day. Nevertheless, these pattern differences are subtle, and the impact they may have on the daily activity of F8 Het mice remains to be explored.

In sum, our results suggest that Fgf8 plays a critical role in the development and some functional aspects of the SCN in adulthood. Since circadian rhythm governs the most fundamental physiological functions in vertebrates, the disruption of Fgf8 signaling may have far-reaching impacts on the overall health of organisms. For example, Fgf8 deficiency may also detrimentally impact sleep, metabolism, reproduction, feeding, growth, and aging by secondarily altering the SCN organization (38–43). Since humans harboring *Fgf8* mutations have been identified (44), our results shed light on potential circadian disorders yet to be diagnosed in these afflicted individuals.

## Author Contributions

P-ST, AM, and SK designed the study; AM performed IHC and wrote the manuscript; P-ST and AM edited the manuscript; all authors performed data analysis and approved the manuscript.

## Conflict of Interest Statement

The authors declare that the research was conducted in the absence of any commercial or financial relationships that could be construed as a potential conflict of interest.
